# Proteomic test for anti-PD-1 checkpoint blockade treatment of metastatic melanoma with and without BRAF mutations

**DOI:** 10.1186/s40425-019-0569-1

**Published:** 2019-03-29

**Authors:** Paolo A. Ascierto, Mariaelena Capone, Antonio M. Grimaldi, Domenico Mallardo, Ester Simeone, Gabriele Madonna, Heinrich Roder, Krista Meyer, Senait Asmellash, Carlos Oliveira, Joanna Roder, Julia Grigorieva

**Affiliations:** 1 Istituto Nazionale Tumori IRCCS Fondazione “G. Pascale”, Naples, Italy; 2 Department of Translational Medical Sciences and Center for Basic and Clinical Immunology Research (CISI), University of Naples Federico II, 80131 Naples, Italy; 3 Biodesix Inc, Boulder, CO USA

**Keywords:** Immunotherapy, Proteomic test, Melanoma, *BRAF* mutations, Anti-PD-1, Nivolumab, Pembrolizumab

## Abstract

The therapeutic landscape in metastatic melanoma has changed dramatically in the last decade, with the success of immune checkpoint inhibitors resulting in durable responses for a large number of patients. For patients with *BRAF* mutations, combinations of BRAF and MEK inhibitors demonstrated response rates and benefit comparable to those from immune checkpoint inhibitors, providing the rationale for sequential treatment with targeted and immunotherapies and raising the question of optimal treatment sequencing.

Biomarkers for the selection of anti-PD-1 therapy in *BRAF* wild type (*BRAF* WT) and in *BRAF* mutated (*BRAF* MUT) patients help development of alternative treatments for patients unlikely to benefit, and might lead to better understanding of the interaction of checkpoint inhibition and targeted therapy. In this paper we evaluate the performance of a previously developed serum proteomic test, BDX008, in metastatic melanoma patients treated with anti-PD-1 agents and investigate the role of *BRAF* mutation status. BDX008, a pre-treatment proteomic test associated with acute phase reactants, wound healing and complement activation, stratifies patients into two groups, BDX008+ and BDX008-, with better and worse outcomes on immunotherapy.

Serum samples were available from 71 patients treated with anti-PD1 inhibitors; 25 patients had *BRAF* mutations, 39 were wild type. Overall, BDX008+ patients had significantly better overall survival (OS) (HR = 0.50, *P* = 0.016) and a trend for better progression-free survival (PFS) (HR = 0.61, *P* = 0.060) than BDX008- patients. BDX008 classification was statistically significant in the analyses adjusted for mutation status, LDH, and line of treatment (*P* = 0.009 for OS and 0.031 for PFS). *BRAF* WT BDX008+ patients had markedly long median OS of 32.5 months and 53% landmark 2 years survival, with statistically significantly superior OS as compared to BDX008- patients (HR = 0.41, *P* = 0.032). The difference between BDX008+ and BDX008- in PFS in *BRAF* WT patients and in OS and PFS in *BRAF* MUT patients did not reach statistical significance, though numerically was consistent with overall results. The test demonstrated significant interaction with neutrophil-to-lymphocyte ratio (NLR) (PFS *P* = 0.041, OS *P* = 0.004). BDX008 as a biomarker selecting for benefit from immune checkpoint blockade, especially in patients with wild type *BRAF* and in subgroups with low NLR*,* warrants further evaluation.

**Electronic supplementary material:**

The online version of this article (10.1186/s40425-019-0569-1) contains supplementary material, which is available to authorized users.

## Background

Remarkable progress in treatment of metastatic melanoma patients in the past decade led from only marginal survival benefit from chemotherapy, which was a standard of care before 2011 [1], to 20–30% of durable responses and approximately 42–47% 3 years survival in advanced patients harboring *BRAF* mutations treated with targeted therapy [2, 3]. In unselected patients treated with immune checkpoint inhibitors landmark 4 years survival was 46–53% [4, 5] with durable antitumor immunity persistent 2 years after the cessation of treatment [6].

Approximately 40–50% of patients with metastatic cutaneous melanoma harbor *BRAF* V600 mutations, which constitutively activate the mitogen-activated protein kinase (MAPK) pathway. The BRAF inhibitors vemurafenib and dabrafenib had shown high response rates in this group of patients. Addition of downstream MEK inhibitors, such as trametinib or cobimetinib, to BRAF inhibitors, resulted in improvements in efficacy over monotherapy, with median PFS of approximately 12 months and around 20% of patients remaining progression-free for 3 years. The new combination of encorafenib and binimetinib resulted in improved median PFS (15 months) and OS (34 months) and 3 years progression-free survival in 28% of patients [3].

Monoclonal antibodies against cytotoxic T-lymphocyte antigen-4 (CTLA-4) and programmed cell death protein 1 (PD-1) and its ligand (PD-L1) have demonstrated high activity in melanoma and other solid tumors. Ipilimumab was the first FDA-approved anti-CTLA-4 agent to achieve superiority against dacarbazine and 20% survival at 3 years and up to 10 years [7, 8]. Anti-PD-1 antibodies demonstrated good clinical activity with less toxicity than chemotherapy or ipilimumab [9, 10]. The combination of nivolumab and ipilimumab has shown superior activity over monotherapy with either nivolumab or ipilimumab in previously untreated patients, independent of *BRAF* status [4], however at the cost of more grade 3 or 4 adverse events.

Advanced melanoma *BRAF* MUT patients receiving the newest combinations of BRAF and MEK inhibitors achieved outcomes similar to those of unselected patients on ipilimumab/nivolumab therapy [11]; however, immunotherapy resulted in more patients remaining progression-free in the long term. While in the *BRAF* WT population immunotherapy has become a standard of care, an optimal strategy in patients with *BRAF* mutations is not that clear. Preclinical evidence suggested a synergistic effect from a combination of targeted and immunotherapies due to activation of the immune system by BRAF/MEK inhibitors and showed promising efficacy in clinical settings [12], however was hindered by a high toxicity rate [13]. Sequential treatment with immune checkpoint and BRAF/MEK inhibitors is considered more suitable for broad clinical practice; several ongoing prospective clinical trials are comparing different sequential approaches (NCT02631447, NCT02224781). It was proposed that tumors innately resistant to anti-PD-1 therapy share a transcriptional signature with melanoma cells treated with MAPK inhibitors [14]; however, it is not yet known whether a shared phenotype predicting sensitivity to BRAF inhibitors and anti-PD-1 agents exists. It would be ideal to find a test identifying either responders to targeted therapy who are unlikely to benefit more from immunotherapy or patients who may be good candidates for more aggressive treatment, such as triplet combination in patients with *BRAF* mutations [12]. Since BRAF and MEK inhibitors have a modifying effect on the tumor microenvironment [15], testing should be done before each new therapy type, and it is important to validate that a particular molecular test is applicable to patients with different treatment histories. It would also be advantageous to have a test that does not rely on tissue availability and could be repeated in the course of multiple lines of treatment.

The BDX008 test was developed to identify patients with better or worse outcomes when treated with immune therapies, using a cohort of ipilimumab-naïve and ipilimumab-pretreated patients from the NCT01176461 clinical study [16, 17]. The test uses matrix-assisted laser/desorption ionization (MALDI) mass spectrometry to measure the circulating proteome in blood; it requires a minimal amount of pre-treatment serum (< 10 μL). BDX008 has been previously validated in several independent cohorts in melanoma and lung cancers [17, 18].

Given the potential clinical utility of BDX008 for optimization of advanced melanoma treatment, we sought to further validate the test in an independent cohort of patients with known *BRAF* mutation status treated with anti-PD-1 therapy in an unselected population previously treated with ipilimumab. Considering that the majority of *BRAF* MUT patients had received BRAF and/or MEK inhibitors in prior lines, we were interested to see whether the performance of the test would be different in this subgroup of melanoma patients. In additional exploratory analysis we have evaluated the effect of the BDX008 test depending on neutrophil-to-lymphocyte ratio (NLR), which is a surrogate marker of systemic inflammation [19] and is known to be prognostic for outcomes in melanoma and other solid tumors [20].

## Methods

### Patients and samples

In this retrospective observational study, 71 pre-treatment serum samples from patients receiving anti-PD-1 therapy were available for analysis and passed quality control in the generation of mass spectra.

Patients were treated with nivolumab at 3 mg/kg every 2 weeks or pembrolizumab at 2 mg/kg every 3 weeks until progression or occurrence of toxicity. One patient had received no prior treatment; the rest of the patients were pre-treated with ipilimumab at 3 mg/kg every 3 weeks for 4 cycles. 24% of patients were treated with immune checkpoint inhibitors in 2nd line, 75% in 3rd or higher line. 55% of patients were *BRAF* WT, 35% were *BRAF* MUT, and for 10% the *BRAF* status was not available. 88% of *BRAF* MUT patients had received prior treatment with vemurafenib (960 mg b.i.d.) and/or cobimetinib (60 mg/die for 21 days every 4 weeks).

Patient characteristics are summarized in Additional file 1: Table S1. Patient characteristics for the whole cohort; individual clinical data and outcomes are described in Additional file 2: Clinical information and outcomes.

### Spectra acquisition and processing

Samples were processed in the same way as for the development of BDX008 using standardized operating procedures described in detail in the Supplementary materials (Additional file 3).

### BDX008 test

BDX008 was previously developed using modern machine learning techniques, optimized to minimize potential for overfitting and maximize generalization to unseen data sets in cases when there are more attributes measured than samples available. One hundred nineteen samples from patients with unresectable melanoma treated with nivolumab in the scope of the NCT01176461 clinical trial were used in test development [17] (details can be found in the Supplementary materials, Additional file 3). BDX008 stratifies patients into two groups, BDX008+ and BDX008-, with better and worse outcomes on immunotherapy.

The test was applied without changes to the described cohort of advanced melanoma patients blinded to clinical data.

### Statistical analysis

All analyses were performed using SAS9.3, SAS Enterprise Guide (SAS Institute, Cary, NC) or PRISM (GraphPad, La Jolla, CA).

Survival plots and medians were generated using the Kaplan-Meier method. Hazard ratios and *P* values were calculated using Cox proportional hazard models; all P values are two-sided.

## Results

Thirty patients (42%) were classified as BDX008+ and 41 (58%) were classified as BDX008-. Patient characteristics by BDX008 classification for the whole cohort and for subgroups with and without *BRAF* mutations are presented in Table 1. There was no significant correlation of BDX008 classification with *BRAF* status (Fisher’s test *P* = 0.605) and other clinical characteristics. However BDX008- classification was correlated with higher levels of lactate dehydrogenase (LDH), (Fisher’s test *P* = 0.006 at LDH cutoff of twice the upper limit of normal (ULN^1^)) and with NLR ≥5 (Fisher’s test *P* = 0.003).

**Table 1:** Patient characteristics by BDX008 classification for all patients and by *BRAF* status

	All patients (*N* = 71)		*BRAF WT (N = 39)*		*BRAF MUT (N = 25)*	
	BDX008+ (*N* = 30)	BDX008- (*N* = 41)	BDX008+ (*N* = 15)	BDX008- (*N* = 24)	BDX008+ (*N* = 12)	BDX008- (*N* = 13)
Age						
Median (Range)	59 (28–86)	66 (32–80)	59 (34–86)	61 (44–80)	52 (29–69)	67 (32–78)
Gender, n (%)						
Female	16 (53)	19 (46)	8 (53)	10 (42)	7 (58)	7 (54)
Male	14 (47)	22 (54)	7 (47)	14 (58)	5 (42)	6 (46)
*BRAF* Status, n (%)						
Mutation	12 (40)	13 (32)	0 (0)	0 (0)	12 (100)	13 (100)
Wild Type	15 (50)	24 (59)	15(100)	24(100)	0 (0)	0 (0)
NA	3 (10)	4 (10)	0 (0)	0 (0)	0 (0)	0 (0)
Line of Therapy with anti-PD-1, n (%)						
1st	1 (3)	0 (0)	0 (0)	0 (0)	0 (0)	0 (0)
2nd	4(13)	13 (32)	3 (20)	9 (38)	0 (0)	3 (23)
3rd	17(57)	16 (39)	11 (73)	11 (46)	5 (42)	3 (23)
4th	4 (13)	10 (24)	1 (7)	4 (17)	3 (25)	6 (46)
5th and higher	4 (13)	2 (5)	0 (0)	0 (0)	4 (33)	1(8)
Targeted therapy, n (%)						
No	18 (60)	28 (68)	14 (93)	22 (92)	2 (15)	1 (8)
Yes	12 (40)	13 (32)	1 (7)	2 (8)	11 (85)	11 (92)
Anti-PD-1 agent, n (%)						
Nivolumab	9 (30)	15 (37)	6 (40)	10 (42)	1 (8)	4 (31)
Pembrolizumab	21 (70)	26 (62)	9 (60)	14 (58)	11 (92)	9 (69)
NLR, n (%)						
<5	25 (83)	19 (46)	14 (93)	12 (50)	8 (67)	5 (38)
≥5	5 (17)	22 (54)	1 (7)	12 (50)	4 (33)	8 (62)
LDH						
Median (in IU/l)	402	617	379	525	416	728
NA, n (%)	5 (17)	2 (5)	3 (20)	2 (8)	2 (17)	0 (0)
<2ULN^a^, n (%)	22 (73)	21 (51)	12 (80)	15 (63)	7 (58)	5 (38)
2ULN^a^, n (%)	3 (10)	18 (44)	0 (0)	7 (29)	3 (25)	8 (62)
Melanoma Type, n (%)						
cutaneous	22 (73)	24 (59)	11 (73)	14 (58)	10 (83)	9 (69)
mucosal	2 (7)	1 (2)	2 (13)	1 (4)	0 (0)	0 (0)
SPI	2 (7)	2 (5)	1 (7)	1 (4)	1 (8)	0 (0)
uveal	2 (7)	4 (10)	0 (0)	3 (13)	0 (0)	0 (0)
NA	2 (7)	10 (24)	1 (7)	5 (21)	1 (8)	4 (31)

^a^*ULN* upper limit of normal (333 IU/l)

Median PFS and OS for the whole cohort were 3.2 months and 9.9 months, respectively. Unselected by BDX008, patients with *BRAF* mutations had numerically shorter median PFS: 2.6 months vs 5.1 months and OS: 5.5 months vs 15.7 months, than patients with wild type *BRAF*. The differences, however, were not statistically significant: PFS HR = 1.21 (95% CI: 0.70–2.09), *P* = 0.487; OS HR = 1.37 (95% CI: 0.76–2.46), *P* = 0.291. Of note is that patients with *BRAF* mutations tended to be treated with the anti-PD-1 agent in higher lines (average/median number of lines 2.8/3 in *BRAF* WT and 3.7/4 in *BRAF* MUT patients, Fisher’s test *P* = 0.076 for patients in 2nd line vs. higher lines). Three of the BRAF WT patients had uveal melanoma; with these patients excluded BRAF WT patients had median PFS and OS of 8.0 months and 16.4 months, respectively. The difference in PFS and OS between non-uveal *BRAF* WT and *BRAF* MUT patients remained not statistically significant (data not shown).

**Fig. 1:**
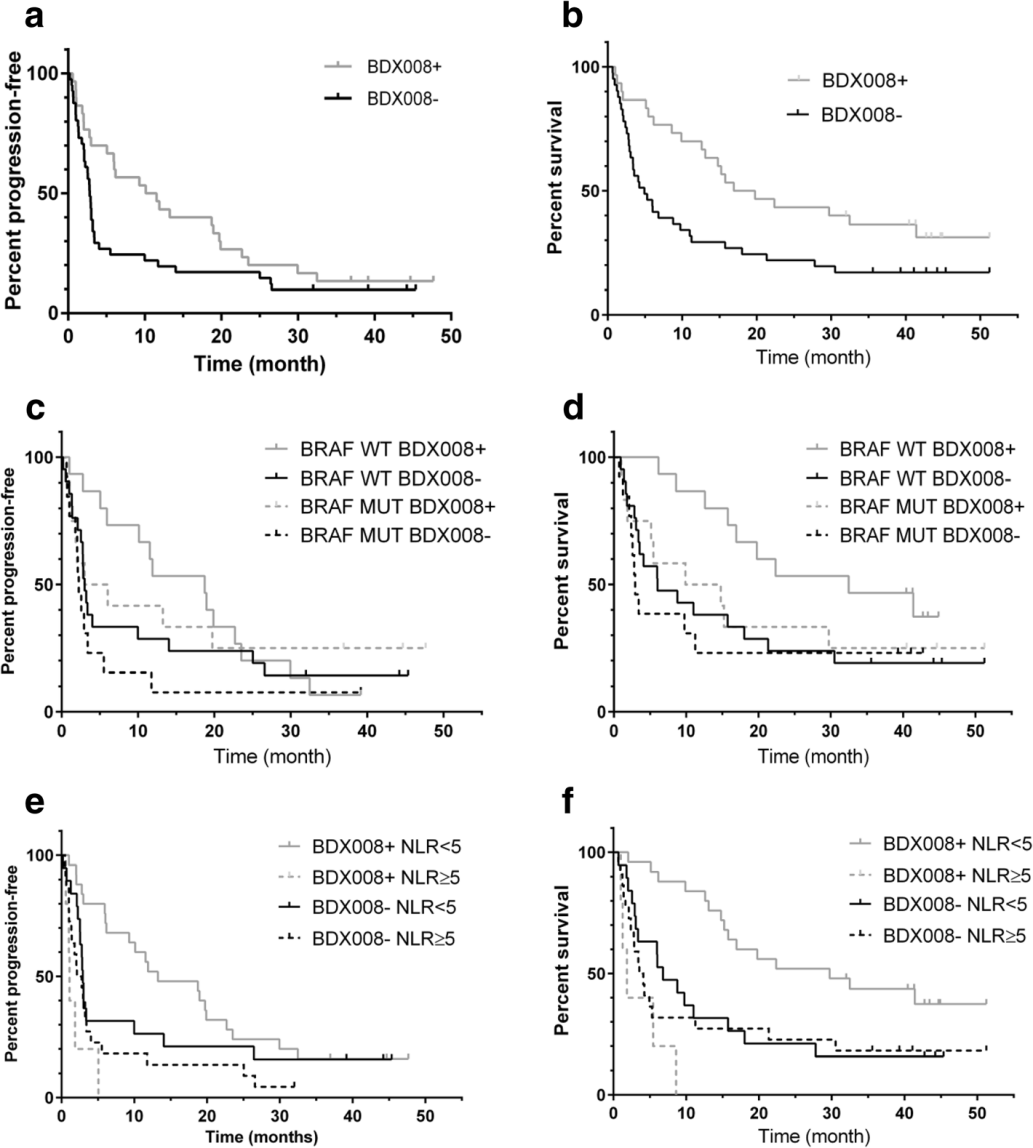
Kaplan-Meier plots of outcome data by BDX008 classification for the whole cohort (**a**-**b**), for patients in subgroups with known *BRAF* status (**c**-**d**), and for patients in subgroups defined by NLR (**e**-**f**)

BDX008+ classification was correlated with best overall response (*P* = 0.005), objective response rate (*P* = 0.056, trend), and disease control rate (*P* = 0.002) (details in Additional file 1: Table S2). OS and PFS results stratified by BDX008 are summarized in Table 2 and Fig. 1. In the whole cohort (*N* = 71), patients classified as BDX008+ had longer PFS and OS than BDX008- patients: median PFS 10.8 vs 2.8 months, HR = 0.61 (95% CI: 0.37–1.02), *P* = 0.060; median OS 18.3 vs 4.9 months, HR = 0.50 (95% CI: 0.29–0.88), *P* = 0.016. Similar results were observed in the *BRAF* WT (not-uveal) (*N* = 36) subgroup for OS: *BRAF* WT BDX008+ patients had significantly better OS than *BRAF* WT BDX008- patients (HR = 0.41 (95% CI: 0.18–0.93), *P* = 0.032); median OS was 32.5 months and 6.0 months in *BRAF* WT BDX008+ and BDX008- patients, respectively. In PFS the separation was not statistically significant: median PFS was 18.7 months in BDX008+ vs 3.0 months in BDX008- patients (HR = 0.70 95% CI: 0.35–1.42), *P* = 0.321) (Table 2 B). Outcome differences in patients with *BRAF* MUT (*N* = 25) with respect to BDX008 classification were not statistically significant (PFS HR = 0.55 (95% CI: 0.23–1.36), *P* = 0.196; OS HR = 0.73 (95% CI 0.29–1.80), *P* = 0.489), though numerically BDX008+ *BRAF* MUT patients had better outcomes than BDX008- *BRAF* MUT patients: median PFS was 4.5 months vs 2.2 months, median OS was 12.3 months vs 2.9 months, respectively (Table 2 C). It appears that *BRAF* WT patients classified as BDX008+ have especially good outcomes on anti-PD-1 treatment, while patients classified as BDX008- have poor prognosis on immunotherapy independently of their mutation status (Fig. 1, c-d). In the exploratory analysis, when patients were stratified by NLR, BDX008+ classification was associated with improved OS in the low NLR subgroup (*N* = 44): median OS was 29.7 months vs 6.8 months in BDX008+ and BDX008-, respectively, HR = 0.38 (95%CI: 0.19–0.79, *P* = 0.008) (Table 2 D, Fig 1 e-f); BDX008+ patients also had numerically higher PFS (13.2 months vs 2.9 months in BDX008-, *P* = 0.169). In the high NLR subgroup (N = 25) BDX008 classification was not associated with PFS or OS (Table 2 E, Fig. 1 e-f).

**Table 2:** Treatment outcomes by test classification for the overall population (A), *BRAF* WT and *BRAF* MUT subgroups (B, C); and NLR < 5 and NLR ≥ 5 subgroups (D, E)

Classification	PFS		OS	
	BDX008-	BDX008+	BDX008-	BDX008+
A: All patients (*N* = 71)				
2 years PFS or OS	17%	20%	22%	43%
Median, months (95% CI)	2.8 (2.1–3.2)	10.8 (5.1–19.7)	4.9 (2.9–9.8)	18.3 (12.6–41.4)
HR – vs + (95% CI)	0.61 (0.37–1.02)		0.50 (0.29–0.88)	
P value	0.060		0.016	
B: BRAF WT, excluding uveal (N = 36)				
2 years PFS or OS	24%	20%	24%	53%
Median, months (95% CI)	3.0 (2.1–9.99)	18.7 (5.1–22.7)	6.0 (3.1–18.0)	32.5 (12.6-Undf*)
HR – vs + (95% CI)	0.70 (0.35–1.42)		0.41 (0.18–0.93)	
*P* value	0.321		0.032	
C: BRAF MUT (N = 25)				
2 years PFS or OS	8%	25%	23%	33%
Median, months (95% CI)	2.2 (0.99–3.4)	4.5 (0.99-Undf)	2.9 (2.1–11.2)	12.3 (1.25-Undf)
HR – vs + (95% CI)	0.55 (0.23–1.36)		0.73 (0.29–1.80)	
*P* value	0.196		0.489	
D: NLR < 5 (*N* = 44)				
2 years PFS or OS	21%	20%	21%	52%
Median, months (95% CI)	2.9 (2.2–10.0)	13.2 (6.2–22.7)	6.8 (2.9–15.7)	29.7 (15.2-Undf)
HR – vs + (95% CI)	0.63 (0.33–1.22)		0.38 (0.19–0.79)	
*P* value	0.169		0.008	
E: NLR ≥5 (N = 27)				
2 years PFS or OS	14%	0%	23%	0%
Median, months (95% CI)	2.4 (1.0–3.4)	1.1 (0.6–5.1)	3.8 (2.1–11.2)	1.8 (0.99–8.6)
HR – vs + (95% CI)	1.88 (0.69–5.14)		1.80 (0.65–5.00)	
*P* value	0.220		0.255	

*Undf = Undefined

In multivariate analyses adjusted for mutation status, line of treatment, and LDH level, BDX008 classification remained significantly associated with PFS and OS (*P* = 0.031 and 0.009, respectively); high LDH and higher line of treatment (> 2) were also significantly associated with worse outcomes (*P* = 0.027 and 0.011 for LDH and *P* = 0.016 and 0.008 for line of treatment for PFS and OS, respectively), while *BRAF* mutations were not significant (*P* = 0.895 and 0.793 for PFS and OS, respectively), see Table 3, A. In the multivariate analysis of OS that included all these factors plus NLR as variables, line of treatment and LDH remained significant (*P*=0.010 and 0.008, respectively), while BDX008 and NLR trended towards significance (*P* = 0.097 and 0.094, respectively (Additional file 1: Table S3). The Kaplan-Meier plots (Fig. 1 e-f) suggested that the effect may be qualitatively different in the subgroups. Indeed, the analysis of interaction between BDX008 classification and NLR (Table 3 B) was significant both in PFS (*P* = 0.041) and OS (*P* = 0.004), confirming the importance of both variables for prognosis.

**Table 3:** Multivariate analysis of PFS and OS (A) – un-stratified, (B) – stratified by treatment line, including interaction of NLR and BDX008

	PFS		OS	
	P	HR (95% CI)	P	HR (95% CI)
A				
BDX008 (+ vs -)	0.031	0.51 (0.28–0.94)	0.009	0.42 (0.21–0.81)
BRAF (MUT vs WT)	0.895	0.96 (0.50–1.85)	0.793	1.09 (0.56–2.14)
Line^a^ (> 2 vs 2)	0.016	2.35 (1.17–4.71)	0.008	3.01 (1.33–6.82)
LDH (high^b^ vs low)	0.027	2.26 (1.10–4.67)	0.011	2.60 (1.24–5.43)
LDH (n/a vs low^b^)	0.322	1.53 (0.66–3.56)	0.332	1.56 (0.63–3.86)
B				
BDX008 (+ vs -)	0.096	0.57 (0.29–1.11)	0.002	0.32 (0.15–0.66)
NLR ≥5 vs < 5	0.183	1.57 (0.81–3.03)	0.592	1.20 (0.61–2.37)
BDX008*NLR interaction	0.041	3.57 (1.06–12.03)	0.004	6.53 (1.82–23.45)

^a^Line of anti-PD-1 therapy

^b^High LDH > 2 ULN, Low LDH < 2 ULN

## Discussion

The goal of this retrospective study was to validate the previously developed BDX008 test in melanoma patients treated with anti-PD-1 therapy and to evaluate its role depending on *BRAF* mutation status. The results confirmed the prognostic property of the test in the whole cohort and in the *BRAF* WT patients, while in the *BRAF* MUT subgroup, numerical advantage in PFS and OS of patients with BDX008+ classification did not reach statistical significance. Importantly, multivariate analysis confirmed that BDX008 classification was significantly associated with PFS and OS independently of *BRAF* status, line of treatment, and LDH; LDH and treatment line were also significant prognostic factors in the analysis, while *BRAF* status was not. Additional exploratory analysis evaluated the effect of the test in relation to NLR - another prognostic factor associated with systemic inflammation. Correlation between high NLR and poor outcomes in melanoma and other solid tumors, including those treated with immunotherapies, was demonstrated previously [20, 21]. We observed a significant interaction between the two factors in PFS and OS; it appears that in patients with BDX008+ classification NLR plays an important role, while in patients with poor prognosis by BDX008, PFS and OS are similar for high and low NLR patients (Fig. 1 e-f). Considering that patients with low NLR and BDX008+ had landmark 2 years survival of 52% and median OS of 28.7 months in advanced lines of treatment with immunotherapy, further studies of using these two biomarkers in combination are warranted.

Small sample size, especially in the *BRAF* MUT subgroup (*N* = 25) and NLR ≥ 5 (*N* = 27), is a significant limitation of this study, resulting in reduced power of the statistical analysis and preliminary nature of the results. Another limitation is a difference in prior therapy and number of prior treatments between the subgroups, which allows only for qualitative comparisons. However, the results appear to be consistent and could be of clinical relevance. Overall, patients with *BRAF* mutations had worse outcomes than *BRAF* WT patients, which could be due to a combination of several factors, such as more previous lines of therapy or poorer sensitivity to anti-PD-1 agents of these patients, when used after treatment with BRAF and MEK inhibitors. Additionally, prior targeted therapy may select for more aggressive disease which is harder to treat, possibly leading to worse outcomes. The multivariate analysis indicated that the line of treatment, rather than *BRAF* mutation status, is significantly correlated with outcomes; however, the majority of patients with *BRAF* mutations had, on average, more lines of treatment and 88% of them were treated with BRAF and /or MEK inhibitors in prior lines.

An important aspect of the BDX008 test is that, instead of focusing on few known molecular markers, BDX008 is a truly multivariate classifier, utilizing information pertinent to the circulating proteome in an unbiased, hypothesis-free way (for details, see Supplementary materials, Additional file 3), tending to reflect the systemic host response to the disease. Subsequent analysis of correlations between test classifications and various biological functions can provide insights into the mechanisms of sensitivity and resistance associated with the test. By applying a gene set enrichment analysis (GSEA) approach [22] to protein expression data, the BDX008 test was shown to be associated with acute phase reactants, wound healing, and complement activation [17]. Independent studies have demonstrated that complement activation can downregulate adaptive antitumor immunity [23], while chronic inflammation, characterized by pathological activation of wound healing processes and up-regulation of various acute phase reactants, creates a tumor-supportive and immune-suppressive microenvironment by activating MAPK pathways, affecting secretion of cytokines, and influencing innate and adaptive immune cells [24]. Observation of significant interaction between NLR and BDX008 in our study is intriguing, because it indicates that while both factors are related to systemic inflammation, they are not equivalent and capture different aspects of the state of the organism, which merits further exploration.

Up-regulation of inflammatory/acute response processes in treatment-naïve patients, as well as a result of prior therapies, including with BRAF/MEK inhibitors, may be part of the biological mechanism related to the poor prognosis associated with the BDX008- classification and with differences in the performance of the BDX008 test in population subgroups, which appears to work better in patients without *BRAF* mutations and in patients with low NLR. However, the effect of smaller sample size in the subgroups, resulting in diminished power of the analysis and, consequently, in the lack of significance in differences between BDX008- and BDX008+ in PFS and OS, also cannot be excluded. Hence, a larger study is needed to confirm the difference between the performance of the test in *BRAF* WT and *BRAF* MUT populations, and to elucidate whether it is defined by the biological aspects related to *BRAF* status or to prior therapy. Notably, patients classified as BDX008+ who were *BRAF* WT or had low NLR, demonstrated especially good outcomes, with median OS exceeding 32 months and 53% landmark 2 years survival, highlighting the clinical utility of the test for predicting good prognosis on anti-PD-1 monotherapy in these groups of advanced melanoma patients.

## Conclusions

In conclusion, this study independently validated previous results that BDX008 stratifies melanoma patients treated with anti-PD-1 agents into groups with better and worse PFS and OS in a mutation-unselected population and in *BRAF* WT patients; its role in patients with *BRAF* mutations and in relation to prior treatments needs to be confirmed in larger patient cohorts. In this study, treatment with anti-PD-1 of *BRAF* MUT patients who were classified as BDX008- resulted in numerically worse outcomes; BDX008+ patients had generally better prognosis, and BDX008+ *BRAF* WT patients had the best outcomes. In patients unselected by BRAF status, the best outcomes were observed in patients with low NLR and BDX008+ classification, indicating the possibility of further refinement of therapy using two biomarkers. However the small sample size and retrospective nature of the study requires further validation of these findings.

## Additional files

Additional file 1: Additional tables. (DOCX 21 kb)

Additional file 2: Clinical information and outcomes. (XLSX 27 kb)

Additional file 3: Supplementary material. (DOC 84 kb)

## Abbreviations

CTLA-4: Cytotoxic T-lymphocyte antigen-4
HR: hazard ratio
LDH: lactate dehydrogenase
MAPK: mitogen-activated protein kinase
NLR: neutrophil-to-lymphocyte ratio
OS: overall survival
PD-1: programmed cell death protein 1
PD-L1: programmed cell death protein 1 ligand
PFS: progression-free survival
ULN: upper limit of normal
