# Supplementary material for: Proteomic test for anti-PD-1 checkpoint blockade treatment of metastatic melanoma with and without BRAF mutations
Source: J Immunother Cancer. 2019 Mar 29;7:91. doi: 10.1186/s40425-019-0569-1 (PMC6440152; doi:10.1186/s40425-019-0569-1)
Supplement: Supplementary file 1 — Additional tables. (DOCX 21 kb) [file 40425_2019_569_MOESM1_ESM.docx]

**Additional Table 1: Patient characteristics for the whole cohort**

|  | Whole Cohort (N = 71) |
| --- | --- |
| **Age** |  |
| Mean (SD) | 60 (13) |
| Median (Range) | 61 (28-86) |
| **Gender, n (%)** |  |
| Female | 34 (48) |
| Male | 37 (52) |
| **BRAF Status, n (%)** |  |
| Mutation | 25 (35) |
| Wild Type | 39 (55) |
| NA | 7 (10) |
| **Line of Therapy with anti-PD1, n (%)** |  |
| 1st | 1 (1) |
| 2nd | 17 (24) |
| 3rd | 33 (46) |
| 4th | 13 (18) |
| 5^th^ | 5 (7) |
| 6th | 2 (3) |
| **Prior Targeted therapy** |  |
| No | 46 (65) |
| Yes | 25 (35) |
| **Anti-PD1 agent** |  |
| Nivolumab | 24 (34) |
| Pembrolizumab | 47 (66) |
| **Prior Ipilimumab** |  |
| No | 1 (1) |
| Yes | 70 (99) |
| **NLR,** n (%) |  |
| <5 | 44 (62) |
| ≥5 | 27 (38) |
| **LDH** |  |
| Median (range) in IU/l | 492 (212-6886) |
| NA, n (%) | 7 (10) |
| <ULN*, n (%) | 10 (14) |
| <2ULN*, n (%) | 43 (61) |
| >2ULN*, n (%) | 21 (30) |
| **Melanoma Type, n (%)** |  |
| cutaneous | 46 (65) |
| mucosal | 3 (4) |
| SPI | 4 (6) |
| uveal | 6 (8) |
| NA | 12 (17) |

^*^ULN= upper limit of normal (333IU/l)

**Table 2: Response to treatment by RECIST for the whole cohort by BDX008 classification**

|  | Best overall response | |  |
| --- | --- | --- | --- |
| Response category, n (%) | BDX008+  (N = 30) | BDX008-  (N=41) | P value |
| CR | 3 (10) | 3 (7) | 0.005 |
| PR | 9 (30) | 4 (10) |  |
| SD | 5 (17) | 1 (2) |  |
| PD | 13 (43) | 33 (80) |  |
| Response | 12 (40) | 7 (17) | 0.056 |
| No Response | 18 (60) | 34 (83) |  |
| Disease control | 17 (57) | 8 (20) | 0.002 |
| No disease control | 13 (43) | 33 (80) |  |

Table 3. Multivariate analysis of PFS and OS including NLR

|  | **PFS** | | **OS** | |
| --- | --- | --- | --- | --- |
|  | **P** | **HR (95% CI)** | **P** | **HR (95% CI)** |
| BDX008  (+ vs -) | 0.338 | 0.71 (0.36-1.43) | 0.097 | 0.54 (0.26-1.12) |
| BRAF  (MUT vs WT) | 0.940 | 0.98 (0.51-1.87) | 0.866 | 1.06 (0.54-2.06) |
| Line*  (>2 vs 2) | 0.028 | 2.20 (1.09-4.42) | 0.010 | 2.98 (1.30-6.80) |
| LDH  (high** vs low) | 0.026 | 2.24 (1.10-4.57) | 0.008 | 2.73 (1.31-5.70) |
| LDH  (n/a vs low**) | 0.353 | 1.51 (0.64-3.56) | 0.326 | 1.58 (0.63-3.95) |
| NLR ≥5 vs <5 | 0.035 | 2.0 (1.05-3.79) | 0.094 | 1.76 (0.91-3.43) |

*Line of anti-PD-1 therapy
** High LDH> 2 ULN, Low LDH <2 ULN
