# Supplementary material for: Proteomic test for anti-PD-1 checkpoint blockade treatment of metastatic melanoma with and without BRAF mutations
Source: J Immunother Cancer. 2019 Mar 29;7:91. doi: 10.1186/s40425-019-0569-1 (PMC6440152; doi:10.1186/s40425-019-0569-1)
Supplement: Supplementary file 3 — Supplementary material. (DOC 84 kb) [file 40425_2019_569_MOESM3_ESM.doc]

Ascierto et al., JITC 2018

# Supplementary Material

## Sample Preparation

Samples were thawed and 3 μl aliquots of each test sample and quality control/reference serum (a pooled sample obtained from serum of five healthy patients, “SerumP3”) spotted onto serum cards (Therapak). The cards were allowed to dry for 1 hour at ambient temperature after which the whole serum spot was punched out with a 6mm skin biopsy punch (Acuderm). Each punch was placed in a centrifugal filter with 0.45 µm nylon membrane (VWR).  One hundred μl of HPLC grade water (JT Baker) was added to the centrifugal filter containing the punch. The punches were vortexed gently for 10 minutes then spun down at 14,000 rcf for two minutes. The flow-through was removed and transferred back on to the punch for a second round of extraction. For the second round of extraction, the punches were vortexed gently for three minutes then spun down at 14,000 rcf for two minutes. Twenty microliters of the filtrate from each sample was then transferred to a 0.5 ml eppendorf tube for mass spectral analysis.

All subsequent sample preparation steps were carried out in a custom designed humidity and temperature control chamber (Coy Laboratory). The temperature was set to 30 °C and the relative humidity at 10%.

An equal volume of freshly prepared matrix (25 mg of sinapinic acid per 1 ml of 50% acetonitrile:50% water plus 0.1% TFA) was added to each 20µl serum extract and the mix vortexed for 30 sec. The first three aliquots (3 x 2µl) of sample:matrix mix were discarded into the tube cap. Eight aliquots of 2µl sample:matrix mix were then spotted onto a stainless steel MALDI target plate (SimulTOF).  The MALDI target was allowed to dry in the chamber before placement in the MALDI mass spectrometer.

## Spectra acquisition

Spectra were obtained using a MALDI-TOF mass spectrometer (SimulTOF 100 s/n: LinearBipolar 11.1024.01 from Virgin Instruments, Sudbury, MA, USA). The instrument was set to operate in positive ion mode, with ions generated using a 349 nm, diode-pumped, frequency-tripled Nd:YLF laser operated at a laser repetition rate of 0.5 kHz. External calibration was performed using a mixture of standard proteins (Bruker Daltonics, Germany) consisting of insulin (*m/z* 5734.51 Da), ubiquitin (*m/z*, 8565.76 Da), cytochrome C (*m/z* 12360.97 Da), and myoglobin (*m/z* 16952.30 Da).

Spectra from each MALDI spot were collected as 800-shot spectra that were 'hardware averaged' as the laser fires continuously across the spot while the stage is moving at a speed of 0.25 mm/sec. A minimum intensity threshold of 0.01 V was used to discard any 'flat line' spectra. All 800-shot spectra with intensity above this threshold were acquired without any further processing.

## Spectra processing and feature definition

To allow for deeper and less noisy probing of the serum proteome, Deep MALDI average spectra were created. [Duncan, M. W. Asmellash, S. G., Sayers, K., Tsypin, M., Roder, J., Roder, H. Extending the Information Content of the MALDI Analysis of Biological Fluids (Deep MALDI). Proceedings of the 61st ASMS Conference on Mass Spectrometry and Allied Topics. 2013, MP 181]

Raster spectra were aligned as described in detail in Weber et al. [Weber, J. et al. A Serum Protein Signature Associated with Outcome after Anti–PD-1 Therapy in Metastatic Melanoma. Cancer Immunol Res January 1 2018 (6) (1) 79-86].

For each sample, 800-shot ‘raster’ spectra were then averaged to create the Deep MALDI average spectrum.

Deep MALDI average spectra (further referred as “spectrum”) were further processed to render them reproducible and comparable across samples. The background in each spectrum was estimated and subtracted. Spectra were then normalized in several stages: First, a coarse normalization was performed using a partial ion current (PIC) approach using regions of the spectra that showed low variability across the population of interest and that showed no sign of association with outcomes. Using the normalized spectra, a second finer PIC normalization was carried out after a check on the variability of the normalized spectra and any dependence on outcome. Although the spectral alignment is typically very good, due to the alignment carried out in the raster processing, the spectra were aligned to address minor differences in peak positions that might still be present.

Features were defined in the mass spectra. These were defined by visual inspection of an indication representative set of spectra. A feature is an m/z region in the mass spectrum, specified by its lower m/z limit and its upper m/z limit. While features were defined based on the location of mass spectral peaks in typical spectra, for any individual spectrum the feature may or may not contain a well-defined mass spectral peak. Once the features were defined, they became parameters in the fully-specified test. For each feature and spectrum a feature value was defined as the sum of intensity values of all data points in the spectrum within the feature. For this test 351 features were defined and used in various aspects of processing, although only 59 were used by the classification algorithm.

To ensure that spectral data can be reproducibly generated, the QC/reference samples that were included at the beginning and end of each batch of samples run were used to batch correct the feature values of each batch of samples.

The final step in processing of the spectra was another PIC normalization step. The final normalization coefficient was calculated for each spectrum and all feature values were divided by the normalization coefficient for the spectrum/sample to produce the final processed feature values. These were the feature values that were used for the development set samples for the creation of the test and these would be the feature values that would be input into the test classification algorithm when performing the fully specified test on a new sample.

## BDX008 test development

Samples from 119 patients with unresectable melanoma treated with nivolumab in the scope of the NCT01176461 clinical trial were used in development. The test was designed to stratify patients into two groups: high likelihood of good outcome – which we call “BDX008+”, low likelihood of good outcome – which we call “BDX008-”.

**A hierarchical classifier development platform designed for problems where the number of available instances is smaller than the number of measured attributes**

The classifier was created using a hierarchical classifier development platform designed specifically to work well in settings where the number of attributes (features) measured for each instance (sample) exceeds the number of instances available for classifier training. It incorporates aspects of traditional and modern machine learning, including bagging, boosting, and regularization using dropout, with the aim of producing classifiers with reliable performance estimates from relatively small sample sets while minimizing chances of overfitting to peculiarities in the development set data (A detailed description can be found in US patent 9,477,906 Roder H, Roder J, inventors; Biodesix, Inc., assignee. Classification generation method using combination of mini-classifiers with regularization and uses thereof (2016) and Weber, J. et al. A Serum Protein Signature Associated with Outcome after Anti–PD-1 Therapy in Metastatic Melanoma. Cancer Immunol Res January 1 2018 (6) (1) 79-86.)

The set of samples available for development was randomly split into training and test sets many (625) times. This prevented the use of one training/test split that may be particularly easy or hard to classify in testing or particularly poor for training. For each training/test split realization, many classifiers (“atomic classifiers”) were built using subsets of the mass spectral features. Here we used k-nearest neighbor classifiers with fixed k=9 for atomic classifiers and used all single features and all possible combinations of pairs of features chosen from a reduced feature subset determined via a bagged feature deselection process (described separately below). Each atomic classifier was applied to its training set and the accuracy of the resulting classification groups assessed. The atomic classifiers were filtered so that only classifiers demonstrating acceptable classification performance were used further in the platform. The performance of the individual atomic classifiers did not need to be excellent, as they were later combined. This approach uses the idea of boosting [R.E. Schapire, “The Strength of Weak Learnability,” Machine Learning, vol. 5, pp. 197-227, 1990] – that many classifiers of decent performance can be combined into an overall classifier with at least as good, or better, performance. The use of many atomic classifiers increases test robustness, adding more protection against overfitting, as we were not selecting the few top performing classifiers or features based on their apparently superior performance on the training set.

Once the atomic classifiers were filtered and poorly performing classifiers eliminated, the remaining atomic classifiers were combined to create one base classifier per training/test split realization. This was done using logistic regression over the training set samples. As there are very many atomic classifiers that pass filtering, it is essential to employ a strong regularizer to avoid overfitting during the regression. We used the concept of dropout, a technique commonly used as the regularizer during the training of deep learning nets [N Srivastava et al, Dropout: A simple way to prevent neural network overfitting. Journal of Machine Learning Research, vol. 15 pp.1929-1958, 2014]. Our regularization method was implemented as follows: From the pool of atomic classifiers passing filtering, we randomly selected 10 atomic classifiers. We performed the logistic regression to calculate weights for combining this subset of atomic classifiers. We repeated this many times, enough that each atomic classifier was drawn many times, each dropout iteration drawing a random set of 10 atomic classifiers. The weight for each atomic classifier was averaged over many dropout iterations to give the weights for the final logistic combination.

The final level of the platform hierarchy was an ensemble average of the base classifiers (bagging over the training/test split realizations) [L. Breiman, Bagging Predictors. Machine Learning. Vol. 24, pp.123-140, 1996]. This was carried out by averaging the output of the logistic function over the bags and then applying a threshold of 0.5 to produce a binary classification.

**Training Class Definition and a Semi-Supervised Approach to Simultaneous Refinement of Training Class Labels and Classifier**

This approach used supervised learning, i.e., it was necessary to know the training class labels for the classification problem, in this instance, which samples were from patients group likely to have good outcomes from immune therapy and which were not. It was not *a priori* clear how to unambiguously define good outcomes on therapy from time-to-event data in a way that revealed underlying information in the molecular data. We employed an approach that simultaneously refined training class labels for classifier development at the same time as the classifier itself.

First an educated guess was made for training class labels. The samples were sorted according to patient survival and the patients with the longest survival were assigned to the good prognosis group and the others to the poor prognosis group. Using these class labels, a classifier was constructed using the hierarchical approach explained above. Once the classifier was created, it was used to classify the samples in the development set or subset, with reliable and unbiased classifications obtained using its ‘out-of-bag’ approach [L. Breiman, Out-of-bag estimators. Technical Report. Dept of Statistics, University of California, 1996]. These classifications were used as the training class labels to create a second classifier, which reclassified the development subset samples, producing an iterative process. This typically converged either exactly or apart from very few samples after 10 or fewer iterations. The result was a classifier together with a consistent set of training labels for the development set.

**Bagged Feature Deselection**

Prior to developing each classifier using the hierarchical platform method, the set of 351 available mass spectral features was pruned down to a smaller set by deselecting features with little apparent utility. The classifier development set was randomly split many times into a training set and a holdout set as for hierarchical classifier development. For each of the 351 features a k-nearest neighbor (kNN) classifier (k=9) was created using the training set and this was applied to the training set samples. The classification performance of the kNN classifier on the training set was assessed and if it exceeded a certain threshold, the feature used was added to a list. This was repeated for all features within each training set realization and across many training set realizations. The cumulative list of features thus assembled was then analyzed. Features occurring very few times or not at all in the list were considered not likely to be useful for classification and were discarded to leave a pruned list of features that was then used within the hierarchical classification development platform. This process was repeated as the training class labels were updated in each iteration of the simultaneous training class label and classifier refinement process and the reduced feature set defined in the final iteration was that used within the final test classification algorithm. This contained the 59 mass spectral features listed in the table below.

Mass spectral features used for classification

| Feature mass/charge |
| --- |
| 3110 |
| 3703 |
| 3723 |
| 3755 |
| 3776 |
| 3928 |
| 3953 |
| 4050 |
| 4133 |
| 4756 |
| 4791 |
| 5020 |
| 5068 |
| 5104 |
| 5145 |
| 5550 |
| 5570 |
| 5734 |
| 5762 |
| 5842 |
| 5867 |
| 5889 |
| 5911 |
| 5950 |
| 5997 |
| 6091 |
| 6109 |
| 6170 |
| 6210 |
| 6568 |
| 6860 |
| 6881 |
| 7318 |
| 8391 |
| 8531 |
| 9109 |
| 11446 |
| 11481 |
| 11527 |
| 11686 |
| 11733 |
| 11787 |
| 11835 |
| 11899 |
| 11952 |
| 12003 |
| 13134 |
| 13323 |
| 13721 |
| 13762 |
| 13843 |
| 17033 |
| 18275 |
| 18637 |
| 18729 |
| 18850 |
| 19992 |
| 23357 |
| 23469 |

The test, consisting of completely specified sample preparation, spectral acquisition, spectral processing, and classification algorithm, was locked after development.
